# Nontuberculous Mycobacteria in Saudi Arabia and Gulf Countries: A Review

**DOI:** 10.1155/2017/5035932

**Published:** 2017-02-27

**Authors:** Hawra Al-Ghafli, Sahal Al-Hajoj

**Affiliations:** Department of Infection and Immunity, King Faisal Specialist Hospital and Research Center, Riyadh, Saudi Arabia

## Abstract

Nontuberculous Mycobacteria (NTM) are causing growing health problems worldwide. This is indicated by an increasing amount of scientific reports showing not only well-identified species reemerging but also emergence of new species. The emergence and reemergence of NTM are particularly worrying in developing countries due to scarce published data and improper identification. Here we aimed to examine the main epidemiological aspects and diagnostic challenges associated with NTM in countries of the Gulf Cooperation Council (GCC) and compare these findings to the international arena findings. Data revealed that countries of the GCC are largely dominated by rapidly growing mycobacteria species such as* M. fortuitum* (29%) and* M. abscessus* (17%) with high rate of definitive respiratory diseases. On the other hand, most of the developed countries are dominated by slowly growing mycobacteria such as MAC,* M. kansasii*, and* M. gordonae*. More efforts are needed, however, to gain insights into NTM issues in countries of the GCC.

## 1. Introduction

*Mycobacterium* is a large ancient genus of Actinobacteria; most members of* Mycobacterium* are described as opportunistic organisms widely spread in our planet either as water, soil, or plant inhabitants [1]. Despite the long existence of mycobacteria, the subidentification of approximately 150* Mycobacterium* species had only been possible in the last two decades [2]. This is largely attributed to the emergence of new identification and fingerprinting approaches [3]. These fingerprinting approaches are time and labor efficient and provide accurate and reliable results, unlike prior descriptive bacterial taxonomy approaches.

The majority of identified mycobacterial species exhibit various levels of pathogenic impact towards humans. By far the most recognized pathogenic species in this genus is* Mycobacterium tuberculosis (M. tuberculosis)*.* M*.* tuberculosis* is responsible for the most deadly mycobacterial infectious disease, tuberculosis (TB), with at least 1.5 million reported deaths worldwide [4]. However, the rest of the identified mycobacterial species do not cause TB and hence they are referred to as Mycobacteria Other Than Tuberculosis (MOTT). Other names designated to MOTT include Atypical Mycobacteria (ATM) and Nontuberculous Mycobacteria (NTM).

Most NTM species are aerobic, nonmotile organisms, with rigid and thick cell walls [5]. The thickness of NTM cell wall functions as a natural protective shield against disinfectants and antibiotics [5, 6]. These characteristics enable them to thrive in most natural resources surrounding humans. NTM can be isolated from domestic and animal products as well as artificial built systems, including shower streams and medical devices [7]. This explains the increasing number of reported NTM infections that occur upon direct ingestion, inhalation, and incubation of mycobacterial virulent agents [8].

In the past years, a sharp increase of reported NTM infections has been noted at a global scale in both immunocompromised and immunocompetent individuals [9–16]. The issue of NTM is in particular worrying in developing regions due to scarce published data and improper identification. In addition, a clear and concise view to the main issues and trends regarding NTM in countries of the Gulf Cooperation Council (GCC) is lacking in the literature. To our knowledge, this is the first review in scientific reporting aiming to provide an updated insight into the case of NTM particularly in countries of the GCC (Saudi Arabia, Qatar, United Arabs of Emirates (UAE), Kuwait, Bahrain, and Oman) and comparing it with other sites of the world.

## 2. Clinical Epidemiology of NTM

As per the collected data, the most two geographically distributed species of NTM (defined as those reported in most GCC countries) are* M. avium complex* and* M. kansasii* (Table 1) [17–21].* M. avium complex* and* M. kansasii *were found in Saudi Arabia with the uppermost number of reported clinical isolates compared to Kuwait and Oman (Table 1). In fact, the diversity burden of* M. avium complex* and* M. kansasii* exceeds the Gulf region and covers most regions of the Middle East with a clinical rate of 12.9% and 9.4%, respectively [14, 17–19, 22–29].

On the other hand, the two less distributed, but more clinically predominant, NTM species in the GCC countries (defined as those reported highest in clinical occurrence) are* M. fortuitum*, MAC, and* M. abscessus* (Table 1).* M. fortuitum* and* M. abscessus* were previously reported in Saudi Arabia and Kuwait, with a similar clinical occurrence in both regions.* M. fortuitum* was accountable for approximately 36% and 50% of clinical isolates in Saudi Arabia and Kuwait, respectively. In contrast,* M. abscessus* accounts for at least 21 and 14% of all reported clinical isolates in Saudi Arabia and Kuwait, respectively (Table 1). Additional NTM species reported from Saudi Arabia include* M. gordonae, M. xenopi, M. haemophilum, M. scrofulaceum, M. chelonae*,* M. smegmatis*, and* M. szulgai*. Although most of these NTM species have been reported with a rare clinical frequency, it is unclear whether their clinical relevance will remain very rare or it will take a different clinical pattern in the coming years. The diversity burden of NTM in Saudi Arabia also comprises a novel species, named* M. riyadhense* [30]. The newly identified kind of mycobacteria,* M. riyadhense*, and* M. marinum* were both reported separately in two independent case studies in Bahrain (Table 2) [31, 32]. Correspondingly, multiple case studies as well as unpublished data from Saudi Arabia are indeed showing a growing trend of* M. riyadhense*, with many isolates being identified recently (Table 2).

Nonetheless, as per the data collected from the GCC countries, Saudi Arabia has the highest NTM clinical diversity, with at least 13 highly diversified NTM species. In contrast, in Kuwait and Oman the diversity of clinically reported NTM species reaches to six and four species, respectively. One explanation of this huge difference in the burden of NTM diversity in Saudi Arabia and the rest of Gulf region could be due to a better clinical coverage of NTM in Saudi Arabia compared to the small sample size reported from Kuwait and Oman (14 and 13 clinical isolates, resp.). In line with this explanation, similar diversity trend noted from Saudi Arabia has been also reported from neighbors of the GCC countries, constituting other regions of the Middle East, with various extents, such as that reported from Iran (12 species of NTM) and Turkey (18 species of NTM) [28, 29, 33]. Alternatively, Saudi Arabia might be under higher vulnerability to NTM exposure and infection when compared with other GCC countries. However, with this limited amount of reported clinical data from the Gulf region and the little research contribution of UAE, Qatar, and Bahrain, our insights into NTM discrepancies remain largely lacking and necessitate more epidemiological investigations.

## 3. Clinical Significance of NTM: Pulmonary and Extrapulmonary Cases

In Saudi Arabia, 73 out of 95 NTM clinical isolates were reported previously as respiratory cases [19]. This included approximately 76.8% of all assessed cases between July 2009 and 2010, confirmed mainly in sputum specimen, followed by other sources of specimens such as bronchial wash, lymph node biopsy, and urine [19]. Common pathogens responsible for respiratory cases found in Saudi Arabia were* M. fortuitum, M. abscessus, M. kansasii*, and MAC [19]. The reported relevance of each pathogen in this study was 34.2%, 28.8%, 9.7%, and 6.8%, respectively. Other less clinically relevant pathogens responsible for the reported cases of pulmonary infections include* M. gordonae, M. xenopi, M. celatum, M. asiaticum*, and* M. simiae*. The rate of definitive pulmonary diseases, which are in agreement with the American Thoracic Society/Infectious Disease Society of America (ATS/IDSA) 2007 guideline, was reported as 67%. Interestingly,* M. fortuitum* also contributed largely to the 24 cases of lung colonizers reported by Varghese et al. (2013). Similar insights have been noted from another study in Saudi Arabia. The study examined respiratory specimens of 142 patients; 28% of them were diagnosed with definitive pulmonary NTM infections, caused mainly by MAC and* M. abscessus* [20]. A summary of all respiratory and colonization cases reported so far from Saudi Arabia is outlined in Table 3.

**Figure 1:**
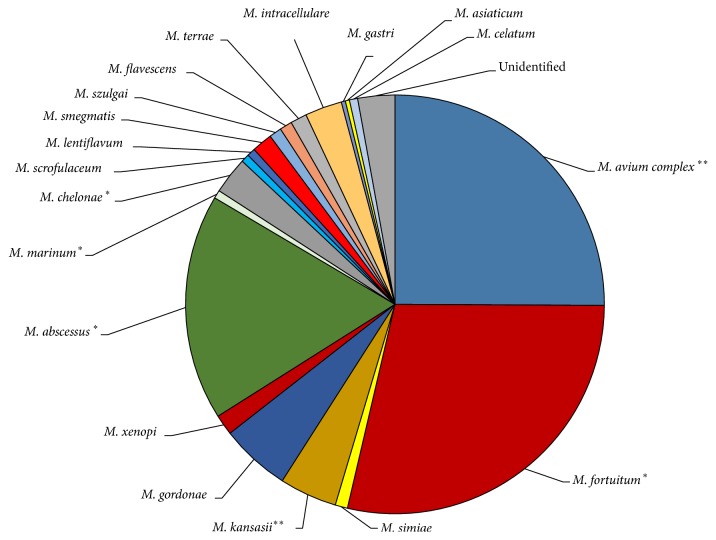
Clinical diversity of NTM in the Gulf countries. Illustrating the clinical diversity of NTM in three different GCC countries: Saudi Arabia, Kuwait, and Oman. The *∗∗* sign is designated for species reported from Saudi Arabia, Kuwait, and Oman. The *∗* sign, however, indicates that a species is reported from two GCC countries (Saudi Arabia and Oman or Saudi Arabia and Kuwait). Species shown in this figure with (no sign) are only reported from one GCC country. References [17–21].

Additionally, Varghese et al. (2013) reported 22 isolates as cases of extrapulmonary NTM infections. Pathogens responsible for these 22 cases include* M. abscessus, M. fortuitum, M. intracellulare, M. gordonae, M. lentiflavum, M. scrofulaceum*, and* M. chelonae*, with various clinical percentages, ranging from 36.4% to 4.9%, respectively (Figure 1) [19], wherein* M. abscessus and M. fortuitum* both contributed predominantly to pulmonary and extrapulmonary cases with high clinical occurrence. In Oman, however, MAC,* M. simiae*, and* M. marinum* were the main etiological agents of NTM pulmonary diseases (Table 3/Figure 1) [17].

Without any doubt, the high occurrence of reported pulmonary NTM infections that meet the ATS/IDSA criteria in Saudi Arabia and Oman (altogether approximately 40%) constitute a serious health issue (Table 3). It remains unclear, however, whether this is the case only in Saudi Arabia and Oman, or there is a bigger trend of high NTM pulmonary infections covering most countries of the GCC. These health incidences, and suspicions of their bigger geographic burden, should be addressed with great deal of attention by health authorities.

## 4. Trends of NTM in Countries of the GCC versus the Rest of the World

**Figure 2:**
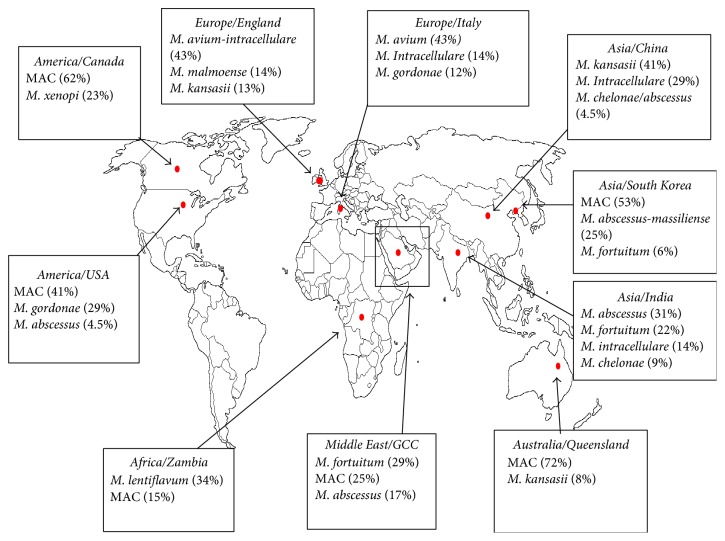
Worldwide geographical distribution of NTM. Showing summarized data of NTM identified from human clinical specimens in different geographical settings [19–21, 34–42, 96].

In general, an increase in NTM clinical isolation has been noted from the Gulf region. Such trend is not surprising and has been echoed in other sites of the world including some countries in Europe, Asia, Australia, and America [34–41]. Interestingly, most developing regions situated in Asia (such as South Korea and India) and the Middle East (such as the GCC countries) reported high prevalence of rapidly growing mycobacteria (RGM) in their clinical settings such as* M. fortuitum* (6–22%) and* M. abscessus* (25–31%) (Figure 2/Table 4) [38, 40]. These species have been noted from the Gulf region with a clinical relevance of 29% and 17%, respectively. In contrast,* M. abscessus* has been identified in China and North America with a clinical prevalence of 4.5% in each case [36, 39].

On the other hand, Europe, North America, and Australia showed significant predominance of slowly growing mycobacteria (SGM) among identified clinical isolates (Table 4) [34–37, 41]. The main SGM detected from the above-mentioned regions were MAC (41–43%),* M. kansasii* (13–41%),* M. gordonae* (12–29%), and* M. malmoense* (14%) [34, 35, 37, 39].

With regard to this notion, it is worth noting that in Europe the clinical isolation of* M. avium-intracellulare* has indeed expanded significantly in the last decade, infecting mainly elderly people [35]. Such profound increase in clinical detection and isolation of MAC species was associated with rapid increase in pulmonary infections [35]. Similar trend has been noted from other sites of Europe, Australia, and North America [34, 36, 37, 41]. Species of MAC were also detected in Asia (India, South Korea, and China), the Middle East (countries of the GCC), and Africa (Zambia) with a clinical rate ranging from 14 to 53% [38–40, 42]. On the other hand, other prevalent SGM (including* M. kansasii* and* M. gordonae*) were evident in the GCC states but with a lower clinical prevalence compared to other industrialized regions.

When it comes to diseases, RGM are usually known to cause cutaneous and osteoarticular infections, while SGM are largely identified as etiological agents for lung and lymph diseases [43]. Thereby, it is rather interesting that in Saudi Arabia* M. fortuitum* and* M. abscessus* together accounted for 53% of the total respiratory cases and 47% of definitive pulmonary infections (Table 3). Similar phenomenon has been noted from India and South Korea.

## 5. Environmental Diversity of NTM

Up to our current time, there is limited number of NTM environmental studies reported from Saudi Arabia and almost no reported environmental isolates from other GCC countries. In Saudi Arabia, Wali et al. (2008) attempted to evaluate the impact of tap water, as a potential source of NTM contamination, on oral cavity of healthy Saudis [44]. The study showed 14/29 isolates as not properly identified and a large proportion of the remaining 15 isolates were identified as* M. gordonae* (53%). Additional water contaminants of NTM include* M. fortuitum* (20%),* M. avium complex* (13%), and* M. kansasii* (13%) [44]. Notably, the study highlights the possibility of contaminated tap water supply in the country, especially by* M. gordonae*, which comes in agreement with similar studies reported elsewhere [45–49]. Furthermore, taking into consideration that the clinical relevance of* M. gordonae* was reported not only from Saudi Arabia but also from Kuwait and Qatar, similar issue of water contamination might be worth further investigation in these two regions (as a potential source of* M. gordonae* infections) [18, 50, 51].

Another study from Saudi Arabia has confirmed the contamination of multiple water dams with NTM in Albaha [52]. A total of 13 dams were entitled for the purpose of this study, with almost 520 isolates being collected (between 20 and 30 July of 2013) from multiple environmental resources such as sands, clay, wet stones, and decayed vegetation. Furthermore, a large proportion (79%) of collected isolates yielded positive results for NTM, which is more than that reported elsewhere [53, 54]. In addition, 145 isolates were carefully selected and subjected to species identification process by Alqumber (2014). Results yielded 11 highly diversified species (ranging from slow to rapid growing mycobacteria). These are as follows:* M. intracellulare*,* M. abscessus*,* M. szulgai*,* M. fortuitum*,* M. avium*,* M. kansasii*,* M. simiae*,* M. gordonae*,* M. terrae *complex,* M. chelonae*, and* M. malmoense* (Table 5).

Alarmingly, these dams are heavily used for human consumption, water recreation, and agriculture use, and accordingly inhabitants of the city (Albaha) are under an accelerating risk for NTM exposure and infection, especially those with immunological diseases. This calls for the relevant health authorities' attention to tackle the issue properly and promptly to reduce exposure to NTM in contaminated water resources. In addition, the high diversity of reported NTM species from water dams in Saudi Arabia supports the notion that water resources can be the main facilitator of NTM exposure and infections in arid and desert climates, such as that seen in Saudi Arabia and other Gulf countries.

**Figure 3:**
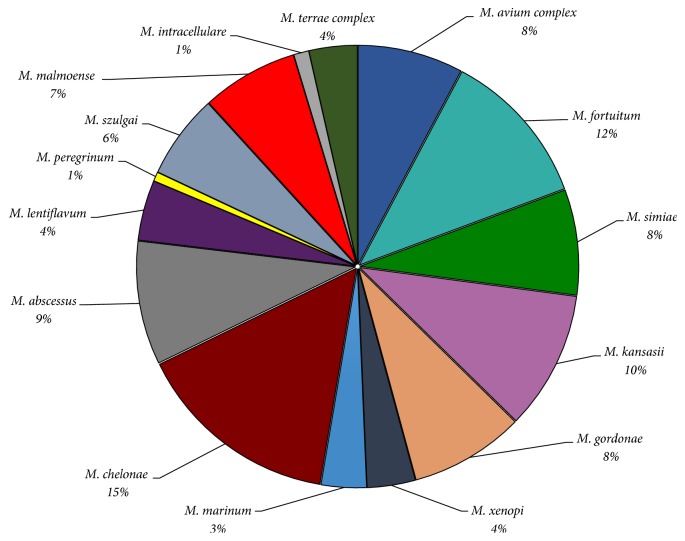
Environmental isolates reported from Saudi Arabia, Iraq, and Turkey. Illustrating the environmental diversity of NTM in Saudi Arabia, Iraq, and Turkey. Species' names and their relevant environmental percentages are also illustrated. References [44, 52, 55–58].

Comparable spectrum of NTM species was reported from other regions of the Middles East and neighbors of the GCC countries, including Iran, Turkey, and Iraq. In Iraq,* M. chelonae* was one of the most common NTM environmental species (18%), isolated from tap water, followed by* M. avium* complex and* M. fortuitum* (Figure 3) [55–57]. The latter two species were also isolated from milk powder and horses feces, respectively [55–57]. Environmental isolates reported from Turkey, however (including* M. lentiflavum*,* M. gordonae*, and* M. peregrinum*), were mainly isolated from hospital water supply [58]. Overall, comprehensive reviews published from Europe, Australia, and Asia as well as the Middle East come in favor of increased rate of pulmonary infections largely caused by MAC species, the most predominant SGM species worldwide, due to contaminated water supply [9, 34, 35, 37, 59]. The scale of the problem, however, remains to be fully defined and characterized in each country of the GCC for more accurate picture.

## 6. Challenges in Diagnosing NTM Diseases 

Diagnosing mycobacterial infections entitles three different stages (clinical, radiological, and microbiological examinations) as per the ATS/IDSA-2007 guideline [8]. In the GCC countries, however, several challenges have been noted during the clinical and microbiological phases. These challenges were addressed accordingly in the following relative sections.

### 6.1. The Need for Optimized Local Diagnostic Criteria

A rapid and accurate recognition of NTM clinical symptoms is indeed a much-desired goal but nonetheless a very challenging one in the Gulf region, not only due to the high diversity of clinical symptoms for each NTM infection or their overlapping features with other diseases, but also due to scarce epidemiological data and the absence of locally optimized diagnostic criteria. The latter two issues make improper identification of NTM a persisting challenge in the Gulf region, as evident from several case studies [30, 60]. Thus, to ensure a prompt recognition of NTM cases in this geographic region, the NTM diagnostic criteria (ATS/IDSA, 2007) should be tested, similar to what has been done elsewhere, and accordingly optimized [61]. This is especially imperative since not all NTM species relevant in countries of the GCC are represented in the ATS/IDSA 2007 criteria. A good example of this is the newly emerging species of* M. riyadhense*. While this species has increased in occurrence during the last years, however, its clinical identification remains a challenge. This emphasizes the need for optimized diagnostic criteria in the region, as well as more epidemiological data from the Gulf countries.

### 6.2. Cross-Contamination and Pseudoinfection

Due to the ambiguity of NTM clinical symptoms, diagnosing NTM diseases relies heavily on the accuracy of laboratories test results. Several factors, however, might undermine the accuracy of laboratories test results and accelerate the frequency of false-positive outcome in the Gulf region. The most two identified factors are (1) cross-contamination and (2) pseudoinfection. Although cross-contamination rate is considered infrequent in most specialized laboratories worldwide, and in some cases inevitable due to human error with a low rate of 3%, it might take place profoundly at below-standards NTM/TB laboratories [62–64]. This issue has been addressed previously concerning some laboratories in the Gulf region [64]. Furthermore, in Saudi Arabia, 22 cases of cross-contamination have been identified through the use of molecular identification techniques, wherein the source of contamination was traced back to a common contaminated buffer [65]. In addition, based on an earlier personal visit to the main TB diagnostic laboratories in Saudi Arabia, in 2004, two main conclusive remarks have been declared [64]. The visited laboratories in Saudi Arabia displayed (1) inadequate laboratories equipment combined with high load of specimens handling and (2) laboratories personnel showed confusion between cross-contamination and other forms of contamination (such as fungus and bacterial contamination) [64]. This raises further speculations of a much bigger relevance of cross-contamination in the country. In addition, better-trained personnel and establishing a standard laboratory protocols were selected as the resolving actions for cross-contamination in countries of the GCC.

On the other hand, unlike the case of cross-contamination, pseudoinfection is more common worldwide and causes a real problem in the issue of accurate NTM diagnosis. Pseudoinfection relies mainly on the resistance capabilities of NTM species towards most disinfectants. This explains their ability to survive on medical devices and laboratory tools used in the diagnosis procedure (such as bronchoscopes) as well as unsterile patients' samples (such as expectorated sputum), causing false-positive NTM diagnosis. Similar cases of pseudoinfection were reported previously in the literature [66]. So far, there is a limited amount of reported cases of pseudoinfection from the Gulf region. It is unclear whether this is due to the scarce amount of available data or due to the insignificant nature of the issue. Furthermore, the 2007 ATS/IDSA guideline offers a practical solution to differentiate pseudoinfection from real NTM diseases. Nonetheless, a better surveillance system must be established to ensure the compliance of physicians and laboratories' technicians with these rules and regulations, especially in local diagnostic laboratories. This may decrease rates of NTM misdiagnosis in the Gulf region and ensure, to some extent, accurate and effective treatments.

## 7. Pathogenicity and Common Risk Factors

Increased predisposition of genetic factors as well as undergoing chronic lung and immune diseases put patients at higher risk of developing NTM infections. In Saudi Arabia, the majority of pulmonary and extrapulmonary cases, reported by Varghese et al. (2013), were observed in patients with previous history of* M. tuberculosis* diseases (PMTD), chronic obstructive pulmonary disorders (COPD), HIV, and cystic fibrosis. The predominant risk factor observed in this study was PMTD (29.5%). In addition, it appears that elderly men in Saudi Arabia are more susceptible to NTM infections as correspondingly reported from another GCC country [18, 19].

In Oman, however, three out of 13 cases have a risk factor to NTM infections. This was due to either heart diseases, HIV, or PMTD, with the same clinical relevance reported for each factor. Thus, the high number of living immunosuppressive patients in the Middle East, comprised of 500,000 people, as well as the high consanguinity rate in some Gulf countries (around 60%), could lead to a drastic increase in NTM infections in the future [67, 68].

While it is true that NTM infections occur predominantly in immunocompromised patients, contradictory cases were also reported from the Gulf region. For instance, pulmonary infections that are caused by* M. abscessus* usually occur to those with genetic predisposition, immunosuppressive, and chronic lung diseases [69, 70]. However, in Saudi Arabia, two healthy individuals, with no genetic and physiological risk factors, were infected with chronic lung diseases caused by* M. abscessus* [71]. These two cases were incorrectly diagnosed as TB before accurate molecular identification. Also, in Kuwait,* M. abscessus* outbreak was responsible for 4 cases of severe infections in a pediatric emergency unit [72].

Notably, the existence of hypervirulent stains of* M. abscessus* in countries of the GCC, compared to the rest of the world, has not been reported before and, in fact, might have been overlooked. To further understand the impact of strain's variation on pathogenicity and drug resistance in countries of the GCC, more research is needed. This should impact positively on the clinical side and may revolutionize the health care system in the region towards precision medicine.

## 8. Emergence of New Species of NTM:* M. riyadhense* as an Example

The debate of newly emergent NTM species has always been linked to recent advancements in fingerprinting and identification techniques. Among many cases of newly identified NTM species,* M. riyadhense* is the only kind that was initially isolated from Saudi Arabia. It was first encountered in a 19-year-old male with a bone infection in left maxillary sinus and with no signs of destructive lung structure. This case was suspected to be bone TB and accordingly the patient was treated with antituberculosis treatment [30]. Later it was found that there are shared genetic virulent factors in* esat-6* and* cfp-10* genes between* M. tuberculosis and M. riyadhense*, with limited understanding to their impact on* M. riyadhense* pathogenicity. Furthermore, the clinical diversity burden of* M. riyadhense* sparked globally after its successful discovery, indicating that it is not geographically contained in Saudi Arabia. It was reported to cause cases of pulmonary infections in Bahrain, France, and Korea as well as three other cases reported recently from Saudi Arabia [60, 73, 74]. Similar to the initial case of* M. riyadhense*, identification of this species was only available through DNA sequencing of certain conserved genes. This underlines the importance of the introduction of* M. riyadhense* in the commercial line-probe assays to ease the rapid identification of* M. riyadhense* and reduce health implications related to late diagnosis worldwide [75]. As shown in the case of* M. riyadhense*, a small percentage of genetic variance (as low as 0.1%) in DNA alignment of well-conserved genes might elucidate the presence of a novel species.

Meanwhile the role of microevolution on bringing about new NTM species cannot be overruled. For instance, evidence of microevolution events was previously reported in a patient infected with TB in the GCC region [76]. In this case study, clonal* M. tuberculosis* variants were identified from a total of five body isolates collected from the same patient: three of them originated from the same site (urine) [76]. Although several studies, published elsewhere, have reported different cases of mixed-TB infections in the same patient, suborgan compartmentalization was rarely reported [77, 78]. It has been proposed by Al-Hajoj et al. (2010) that the long period of infection (associated with a higher rate of mutations) and perhaps genetic drift were the major contributors for the emergence of a subclonal* M. tuberculosis* variant in this patient. Thus, the possibility of similar microevolutionary events, under similar circumstances, in NTM cases should not be entirely excluded. However, there is a trivial research interest on this area in the Gulf region, largely justified by the little amount of solid evidence supporting this theory in encountered clinical cases. In addition, this idea is largely overlooked in NTM research due to assumptions of its small clinical value and accordingly such type of research is unlikely to be supported.

## 9. Conclusion

This review aimed to address the main NTM aspects and challenges in the GCC countries. Although the reported data from the GCC is limited in terms of sample size (most reported papers do not have a nationwide sample size) and geographic coverage (not all countries within the GCC state contribute to the reported data in the literature) it is associated with so far an increasing trend of NTM clinical diversity and severity of infections, with high rate of definitive respiratory diseases. However, more research is required to fill in the recognizable gaps that clearly exist in the literature regarding NTM in countries of the GCC.

## Figures and Tables

**Table 1:** Species distribution of NTM in the Gulf region.

GCC countries						
NTM species	Total number of clinical isolates	Saudi Arabia	Kuwait	Oman	Clinical relevance	Reference
*M. avium* complex	79	68	2	9	Pulmonary	[17–21]
*M. fortuitum *	90	83	7	—	Pulmonary and extrapulmonary	[18–21]
*M. simiae *	3	2	—	1	Pulmonary	[17, 19–21]
*M. kansasii *	14	12	1	1	Pulmonary	[17–21]
*M. gordonae *	17	16	1	—	Pulmonary and extrapulmonary	[18–21]
*M. xenopi *	5	5	—	—	Pulmonary	[19–21]
*M. abscessus*	55	53	2	—	Pulmonary and extrapulmonary	[18–21]
*M. marinum *	2	1	—	1	Pulmonary	[17, 19–21]
*M. chelonae*	9	8	1	—	Extrapulmonary	[18–21]
*M. scrofulaceum *	2	2	—	—	Extrapulmonary	[19–21]
*M. lentiflavum *	2	2	—	—	Extrapulmonary	[19–21]
*M. smegmatis *	5	5	—	—	Pulmonary	[19–21]
*M. szulgai *	3	3	—	—	Pulmonary	[19–21]
*M. flavescens *	3	2	—	1	Pulmonary	[17, 19–21]
*M. terrae *	4	4	—	—	Pulmonary	[19–21]
*M. intracellulare*	9	9	—	—	Pulmonary and extrapulmonary	[19–21]
*M. gastri*	1	1	—	—	Pulmonary	[19–21]
*M. asiaticum*	1	1	—	—	Pulmonary	[19–21]
*M. celatum*	2	2	—	—	Pulmonary	[19–21]
Unidentified	9	9				[19–21]

Total	**315**	**290**	**14**	**13**		

**Table 2:** Case studies reported from the GCC countries.

Country (number of cases)	Species	Number of cases	Site of infection	Source
Saudi Arabia (17)	*M. fortuitum*	4	Pulmonary discharges, ascetic fluid, mediastinal infection, peritoneal dialysis fluid, and lipoid pneumonia.	[79–82]
	*M. abscessus*	4	Pulmonary discharge, peritoneal biopsy, peripheral blood, and permanent catheter tip.	[83–86]
	*M. chelonae*	3	Blood & abnormal fluid, breast abscesses, and pleural fluid.	[87–89]
	*M. marinum*	1	Wound-elbow.	[90]
	*M. kansasii*	1	Appendiceal abscess.	[14]
	*M. szulgai*	1	Joint aspiration.	[91]
	*M. riyadhense*	3	Maxillary sinus, dural lesion, sclerotic lesions, and pulmonary infection.	[30, 60, 74]

Qatar (4)	*M. gordonae*	2	Liver biopsy and urine.	[50, 51]
	*M. fortuitum*	2	Myocardial and abdominal abscess.	[92, 93]

Bahrain (2)	*M. riyadhense*	1	Pulmonary discharge.	[32]
	*M. marinum*	1	Nasal cavity.	[31]

Kuwait (1)	*M. abscessus*	1	Peripheral blood.	[94]

**Table 3:** Pulmonary infections caused by NTM in Saudi Arabia and Oman.

Species	Number of respiratory cases	Number of cases meeting the 2007 ATS criteria	Number of lung colonizers	References
*Saudi Arabia*				
*M. fortuitum*	59	17	42	
*M. abscessus*	45	25	20	
*M. kansasii*	10	8	2	
MAC	64	27	37	
*M. gordonae*	11	2	9	
*M. xenopi*	3	3	0	
*M. celatum*	2	2	0	
*M. szulgai*	2	2	0	
*M. asiaticum*	1	1	0	
*M. simiae*	2	2	0	

Total	199	89 (44.7%)	110 (55.3%)	[19, 20]

*Oman*				
MAC	9	6	3	
*M. kansasii*	1	—	1	
*M. simiae*	1	1	—	
*M. flavescens*	1	—	1	
*M. marinum*	1	1	—	

Total	13	8 (62%)	5 (38%)	[17]

**Table 4:** Summary of the main NTM species identified worldwide.

Region/country	Main NTM species	% of total isolates	NTM diversity	Years	Clinical relevance/comments	Overall trend
Europe/England (review) [35]	*M. avium-intracellulare*	42.9	15 species were identified and two isolates were not properly identified.	1995–2006	Pulmonary and extrapulmonary diseases.	Increase in SGM.
	*M. malmoense*	13.7				
	*M. kansasii*	12.5				
	Others	—				

Europe/Italy (review) [34]	*M. avium*	42.9	13 species were identified, an increasing proportion of unidentified species.	2004–2014	76.2% of clinical isolates were extracted from pulmonary specimens.	Increase in SGM.
	*M. intracellulare*	14.3				
	*M. gordonae*	11.6				
	Others	—				

America/USA (article) [36]	MAC	40.9	22 species were identified.	2001–2009	17% were pulmonary cases and the remaining were due to extrapulmonary infections.	Increase in SGM.
	*M. gordonae*	28.7				
	*M. abscessus*	4.5				
	Others	—				

America/Canada (article) [95]	MAC	62	Not clearly stated but approximately 8 species.	1997–2003 and 2007	Mainly pulmonary infections.	Increase in SGM.
	*M. xenopi*	23				

Australia/Queensland (article) [37]	MAC	72	15 species were identified.	1999–2005	Pulmonary and extrapulmonary infections.	Increase in SGM.
	*M. kansasii*	8				

Asia/China (article) [39]	*M. kansasii*	45	25 species were identified.	2007–2012	Mainly pulmonary cases.	Increase in SGM.
	*M. intracellulare*	20.8				
	*M. chelonae/abscessus*	14.9				
	Others	—				

Asia/South Korea (article) [40]	MAC	53	10 species were identified and 2.5% unidentified.	2001–2011	Mainly pulmonary.	Increase in RGM.
	*M. abscessus-massiliense*	25				
	*M. fortuitum*	6				
	Others	—				

Asia/India (article) [38]	*M. abscessus*	31.3	13 species were identified.	2013–2015	79.8% pulmonary and 18.2% extrapulmonary.	Increase in RGM.
	*M. fortuitum*	22				
	*M. intracellulare*	13.9				
	*M. chelonae*	9.1				
	Others	—				

Middle East/GCC State (articles) [17–21]	*M. fortuitum*	29	A total of 18 species were identified from clinical specimens, though data remain scarce.	1991–2013	Pulmonary and extrapulmonary.	Increase in RGM.
	MAC	25				
	*M. abscessus*	17				
	Others	—				

Africa/Zambia (articles) [42, 96]	*M. lentiflavum*	34	Not very clear but approximately 18 species were identified.	2015 and March to August 2001	Mainly pulmonary.	Not very clear.
	MAC	15				

*M. avium* complex (MAC), Gulf Cooperation Council (GCC), rapidly growing mycobacteria (RGM), and slowly growing mycobacteria (SGM).

**Table 5:** Environmental NTM species identified in Saudi Arabia.

NTM species	Total number of environmental isolates	Source of water contamination	Source
*M. intracellulare*	5	Dams	[52]
*M. abscessus*	8	Dams	[52]
*M. szulgai*	9	Dams	[52]
*M. fortuitum*	15	Dams and tap water	[44, 52]
*M. avium *	14	Dams and tap water	[44, 52]
*M. kansasii*	16	Dams and tap water	[44, 52]
*M. simiae*	15	Dams	[52]
*M. gordonae*	28	Dams and tap water	[44, 52]
*M. terrae* complex	16	Dams	[52]
*M. chelonae*	18	Dams	[52]
*M. malmoense*	21	Dams	[52]
Unidentified species	14	Tap water	[44]

*Total *	*164*		
